# Supplementary material for: Heparin-based hydrogel scaffolding alters the transcriptomic profile and increases the chemoresistance of MDA-MB-231 triple-negative breast cancer cells
Source: Biomater Sci. 2020 Feb 13;8(10):2786–96. doi: 10.1039/c9bm01481k (PMC7497406; doi:10.1039/c9bm01481k)
Supplement: Supplementary file 2 [file BM-008-C9BM01481K-s002.zip › Supplementary File 4/HP-B hydrogel + EGF (s)vControl/Pathways/my_analysis.Gsea.1545201170987/HALLMARK_ESTROGEN_RESPONSE_LATE.html]

Details for gene set HALLMARK\_ESTROGEN\_RESPONSE\_LATE[GSEA]

|  || Dataset | expr.class.cls#HYDEGF\_versus\_CONTROL.class.cls#HYDEGF\_versus\_CONTROL\_repos |
| Phenotype | class.cls#HYDEGF\_versus\_CONTROL\_repos |
| Upregulated in class | CONTROL |
| GeneSet | HALLMARK\_ESTROGEN\_RESPONSE\_LATE |
| Enrichment Score (ES) | -0.32958308 |
| Normalized Enrichment Score (NES) | -1.5171463 |
| Nominal p-value | 0.0 |
| FDR q-value | 0.016158497 |
| FWER p-Value | 0.191 |
Table: GSEA Results Summary

  

Fig 1: Enrichment plot: HALLMARK\_ESTROGEN\_RESPONSE\_LATE      
 Profile of the Running ES Score & Positions of GeneSet Members on the Rank Ordered List

  

| PROBE | DESCRIPTION (from dataset) | GENE SYMBOL | GENE\_TITLE | RANK IN GENE LIST | RANK METRIC SCORE | RUNNING ES | CORE ENRICHMENT || 1 | HOMER2 | na |  |  | 236 | 2.509 | 0.0049 | No |
| 2 | SLC7A5 | na |  |  | 239 | 2.501 | 0.0220 | No |
| 3 | SLC2A8 | na |  |  | 336 | 2.339 | 0.0330 | No |
| 4 | SLC22A5 | na |  |  | 456 | 2.172 | 0.0417 | No |
| 5 | CYP26B1 | na |  |  | 792 | 1.826 | 0.0367 | No |
| 6 | DNAJC1 | na |  |  | 987 | 1.697 | 0.0382 | No |
| 7 | SCARB1 | na |  |  | 994 | 1.690 | 0.0495 | No |
| 8 | XRCC3 | na |  |  | 1015 | 1.676 | 0.0600 | No |
| 9 | ID2 | na |  |  | 1377 | 1.479 | 0.0512 | No |
| 10 | CCNA1 | na |  |  | 1442 | 1.452 | 0.0579 | No |
| 11 | RAPGEFL1 | na |  |  | 1545 | 1.411 | 0.0622 | No |
| 12 | GINS2 | na |  |  | 1691 | 1.361 | 0.0640 | No |
| 13 | TH | na |  |  | 1742 | 1.344 | 0.0706 | No |
| 14 | ITPK1 | na |  |  | 1781 | 1.335 | 0.0778 | No |
| 15 | PKP3 | na |  |  | 1811 | 1.324 | 0.0854 | No |
| 16 | UNC13B | na |  |  | 1911 | 1.290 | 0.0891 | No |
| 17 | FOS | na |  |  | 2215 | 1.207 | 0.0814 | No |
| 18 | PLK4 | na |  |  | 2424 | 1.146 | 0.0784 | No |
| 19 | DLG5 | na |  |  | 2526 | 1.121 | 0.0808 | No |
| 20 | CDC6 | na |  |  | 2609 | 1.102 | 0.0841 | No |
| 21 | FARP1 | na |  |  | 2623 | 1.098 | 0.0910 | No |
| 22 | CCND1 | na |  |  | 2684 | 1.084 | 0.0953 | No |
| 23 | LLGL2 | na |  |  | 2794 | 1.054 | 0.0968 | No |
| 24 | SLC1A4 | na |  |  | 3358 | 0.931 | 0.0737 | No |
| 25 | TSTA3 | na |  |  | 3441 | 0.915 | 0.0756 | No |
| 26 | NCOR2 | na |  |  | 3891 | 0.831 | 0.0578 | No |
| 27 | SIAH2 | na |  |  | 4404 | 0.748 | 0.0360 | No |
| 28 | CDC20 | na |  |  | 4567 | 0.724 | 0.0325 | No |
| 29 | CHPT1 | na |  |  | 4713 | 0.704 | 0.0297 | No |
| 30 | JAK2 | na |  |  | 4877 | 0.671 | 0.0258 | No |
| 31 | AMFR | na |  |  | 4909 | 0.667 | 0.0288 | No |
| 32 | CD44 | na |  |  | 5293 | 0.607 | 0.0128 | No |
| 33 | BCL2 | na |  |  | 5367 | 0.594 | 0.0131 | No |
| 34 | HSPA4L | na |  |  | 5521 | 0.573 | 0.0090 | No |
| 35 | PRKAR2B | na |  |  | 5538 | 0.571 | 0.0121 | No |
| 36 | RBBP8 | na |  |  | 5572 | 0.567 | 0.0142 | No |
| 37 | FGFR3 | na |  |  | 5581 | 0.565 | 0.0177 | No |
| 38 | PTPN6 | na |  |  | 5592 | 0.564 | 0.0211 | No |
| 39 | FRK | na |  |  | 5640 | 0.556 | 0.0224 | No |
| 40 | RABEP1 | na |  |  | 6059 | 0.495 | 0.0039 | No |
| 41 | TFAP2C | na |  |  | 6119 | 0.487 | 0.0041 | No |
| 42 | CELSR2 | na |  |  | 6140 | 0.484 | 0.0064 | No |
| 43 | BATF | na |  |  | 6325 | 0.460 | -0.0001 | No |
| 44 | MYOF | na |  |  | 6482 | 0.438 | -0.0053 | No |
| 45 | PPIF | na |  |  | 6528 | 0.434 | -0.0046 | No |
| 46 | BAG1 | na |  |  | 6712 | 0.409 | -0.0114 | No |
| 47 | SEMA3B | na |  |  | 6755 | 0.403 | -0.0109 | No |
| 48 | NAB2 | na |  |  | 6916 | 0.384 | -0.0166 | No |
| 49 | METTL3 | na |  |  | 6934 | 0.381 | -0.0149 | No |
| 50 | NXT1 | na |  |  | 7070 | 0.363 | -0.0195 | No |
| 51 | ST14 | na |  |  | 7179 | 0.347 | -0.0228 | No |
| 52 | LSR | na |  |  | 7190 | 0.345 | -0.0209 | No |
| 53 | DHRS2 | na |  |  | 7251 | 0.337 | -0.0218 | No |
| 54 | PDZK1 | na |  |  | 7616 | 0.287 | -0.0389 | No |
| 55 | PRLR | na |  |  | 7651 | 0.282 | -0.0387 | No |
| 56 | MICB | na |  |  | 7714 | 0.277 | -0.0401 | No |
| 57 | EGR3 | na |  |  | 7749 | 0.271 | -0.0400 | No |
| 58 | FKBP5 | na |  |  | 7831 | 0.259 | -0.0425 | No |
| 59 | KLF4 | na |  |  | 7914 | 0.254 | -0.0450 | No |
| 60 | KIF20A | na |  |  | 7946 | 0.250 | -0.0450 | No |
| 61 | WFS1 | na |  |  | 7951 | 0.249 | -0.0435 | No |
| 62 | SGK1 | na |  |  | 8039 | 0.239 | -0.0464 | No |
| 63 | CLIC3 | na |  |  | 8093 | 0.230 | -0.0476 | No |
| 64 | MYB | na |  |  | 8130 | 0.230 | -0.0479 | No |
| 65 | HR | na |  |  | 8338 | 0.203 | -0.0574 | No |
| 66 | TNNC1 | na |  |  | 8355 | 0.201 | -0.0568 | No |
| 67 | MAPT | na |  |  | 8425 | 0.198 | -0.0591 | No |
| 68 | STIL | na |  |  | 8443 | 0.196 | -0.0586 | No |
| 69 | ALDH3B1 | na |  |  | 8580 | 0.177 | -0.0646 | No |
| 70 | SNX10 | na |  |  | 8647 | 0.164 | -0.0669 | No |
| 71 | ABCA3 | na |  |  | 8759 | 0.156 | -0.0716 | No |
| 72 | MAPK13 | na |  |  | 8783 | 0.153 | -0.0718 | No |
| 73 | PLXNB1 | na |  |  | 8808 | 0.148 | -0.0720 | No |
| 74 | SFN | na |  |  | 9071 | 0.119 | -0.0850 | No |
| 75 | AFF1 | na |  |  | 9279 | 0.091 | -0.0952 | No |
| 76 | FAM102A | na |  |  | 9501 | 0.075 | -0.1063 | No |
| 77 | TPBG | na |  |  | 9534 | 0.070 | -0.1075 | No |
| 78 | SLC26A2 | na |  |  | 10074 | 0.009 | -0.1358 | No |
| 79 | TPD52L1 | na |  |  | 10368 | -0.032 | -0.1510 | No |
| 80 | ETFB | na |  |  | 10598 | -0.050 | -0.1626 | No |
| 81 | SORD | na |  |  | 10833 | -0.083 | -0.1744 | No |
| 82 | TJP3 | na |  |  | 11329 | -0.139 | -0.1994 | No |
| 83 | CAV1 | na |  |  | 11391 | -0.145 | -0.2016 | No |
| 84 | FOXC1 | na |  |  | 11477 | -0.160 | -0.2050 | No |
| 85 | SLC29A1 | na |  |  | 11581 | -0.172 | -0.2092 | No |
| 86 | FKBP4 | na |  |  | 11609 | -0.176 | -0.2094 | No |
| 87 | MEST | na |  |  | 11757 | -0.197 | -0.2158 | No |
| 88 | PAPSS2 | na |  |  | 11849 | -0.211 | -0.2191 | No |
| 89 | CKB | na |  |  | 12047 | -0.224 | -0.2279 | No |
| 90 | CACNA2D2 | na |  |  | 12305 | -0.262 | -0.2396 | No |
| 91 | JAK1 | na |  |  | 12368 | -0.270 | -0.2410 | No |
| 92 | ADD3 | na |  |  | 12405 | -0.273 | -0.2410 | No |
| 93 | RPS6KA2 | na |  |  | 12594 | -0.297 | -0.2488 | No |
| 94 | TOP2A | na |  |  | 12732 | -0.319 | -0.2538 | No |
| 95 | GALE | na |  |  | 12833 | -0.333 | -0.2568 | No |
| 96 | DCXR | na |  |  | 12972 | -0.336 | -0.2617 | No |
| 97 | RNASEH2A | na |  |  | 13230 | -0.375 | -0.2726 | No |
| 98 | ATP2B4 | na |  |  | 13238 | -0.376 | -0.2704 | No |
| 99 | IL17RB | na |  |  | 13275 | -0.382 | -0.2697 | No |
| 100 | ABHD2 | na |  |  | 13290 | -0.384 | -0.2678 | No |
| 101 | PDLIM3 | na |  |  | 13293 | -0.385 | -0.2652 | No |
| 102 | CDH1 | na |  |  | 13575 | -0.423 | -0.2771 | No |
| 103 | ALDH3A2 | na |  |  | 13652 | -0.434 | -0.2781 | No |
| 104 | GLA | na |  |  | 13717 | -0.444 | -0.2784 | No |
| 105 | ZFP36 | na |  |  | 13904 | -0.472 | -0.2849 | No |
| 106 | IDH2 | na |  |  | 14028 | -0.492 | -0.2880 | No |
| 107 | IGSF1 | na |  |  | 14118 | -0.493 | -0.2893 | No |
| 108 | MOCS2 | na |  |  | 14282 | -0.516 | -0.2943 | No |
| 109 | TST | na |  |  | 14375 | -0.533 | -0.2954 | No |
| 110 | BTG3 | na |  |  | 14693 | -0.589 | -0.3080 | No |
| 111 | ST6GALNAC2 | na |  |  | 15092 | -0.650 | -0.3245 | Yes |
| 112 | TMPRSS3 | na |  |  | 15104 | -0.654 | -0.3205 | Yes |
| 113 | SLC9A3R1 | na |  |  | 15259 | -0.680 | -0.3239 | Yes |
| 114 | OPN3 | na |  |  | 15285 | -0.684 | -0.3205 | Yes |
| 115 | TSPAN13 | na |  |  | 15319 | -0.691 | -0.3175 | Yes |
| 116 | FABP5 | na |  |  | 15412 | -0.703 | -0.3175 | Yes |
| 117 | NBL1 | na |  |  | 15591 | -0.741 | -0.3218 | Yes |
| 118 | ACOX2 | na |  |  | 15595 | -0.742 | -0.3168 | Yes |
| 119 | ARL3 | na |  |  | 15839 | -0.790 | -0.3241 | Yes |
| 120 | TRIM29 | na |  |  | 15879 | -0.798 | -0.3207 | Yes |
| 121 | XBP1 | na |  |  | 16049 | -0.833 | -0.3238 | Yes |
| 122 | FDFT1 | na |  |  | 16053 | -0.834 | -0.3183 | Yes |
| 123 | TIAM1 | na |  |  | 16222 | -0.879 | -0.3210 | Yes |
| 124 | HSPB8 | na |  |  | 16267 | -0.891 | -0.3172 | Yes |
| 125 | SERPINA1 | na |  |  | 16347 | -0.909 | -0.3151 | Yes |
| 126 | DHCR7 | na |  |  | 16459 | -0.938 | -0.3145 | Yes |
| 127 | PRSS23 | na |  |  | 16650 | -0.985 | -0.3177 | Yes |
| 128 | ASS1 | na |  |  | 16742 | -1.009 | -0.3155 | Yes |
| 129 | IMPA2 | na |  |  | 16769 | -1.016 | -0.3099 | Yes |
| 130 | EMP2 | na |  |  | 16919 | -1.061 | -0.3104 | Yes |
| 131 | NMU | na |  |  | 17000 | -1.090 | -0.3071 | Yes |
| 132 | KRT19 | na |  |  | 17022 | -1.096 | -0.3007 | Yes |
| 133 | SERPINA5 | na |  |  | 17287 | -1.177 | -0.3064 | Yes |
| 134 | ELOVL5 | na |  |  | 17447 | -1.235 | -0.3063 | Yes |
| 135 | SCNN1A | na |  |  | 17480 | -1.250 | -0.2994 | Yes |
| 136 | BLVRB | na |  |  | 17508 | -1.262 | -0.2921 | Yes |
| 137 | PTGES | na |  |  | 17682 | -1.324 | -0.2921 | Yes |
| 138 | ISG20 | na |  |  | 17840 | -1.410 | -0.2906 | Yes |
| 139 | AGR2 | na |  |  | 17953 | -1.465 | -0.2864 | Yes |
| 140 | CPE | na |  |  | 18198 | -1.599 | -0.2882 | Yes |
| 141 | TOB1 | na |  |  | 18307 | -1.689 | -0.2823 | Yes |
| 142 | TFF1 | na |  |  | 18353 | -1.713 | -0.2728 | Yes |
| 143 | PLA2G16 | na |  |  | 18422 | -1.773 | -0.2642 | Yes |
| 144 | IL6ST | na |  |  | 18442 | -1.792 | -0.2529 | Yes |
| 145 | HPRT1 | na |  |  | 18443 | -1.794 | -0.2405 | Yes |
| 146 | PDCD4 | na |  |  | 18548 | -1.865 | -0.2331 | Yes |
| 147 | ANXA9 | na |  |  | 18600 | -1.926 | -0.2226 | Yes |
| 148 | LAMC2 | na |  |  | 18772 | -2.148 | -0.2168 | Yes |
| 149 | CISH | na |  |  | 18778 | -2.156 | -0.2022 | Yes |
| 150 | NRIP1 | na |  |  | 18832 | -2.251 | -0.1895 | Yes |
| 151 | AREG | na |  |  | 18856 | -2.288 | -0.1749 | Yes |
| 152 | UGDH | na |  |  | 18907 | -2.368 | -0.1613 | Yes |
| 153 | PERP | na |  |  | 18938 | -2.423 | -0.1462 | Yes |
| 154 | TFPI2 | na |  |  | 18994 | -2.568 | -0.1314 | Yes |
| 155 | RAB31 | na |  |  | 19005 | -2.597 | -0.1140 | Yes |
| 156 | CD9 | na |  |  | 19017 | -2.631 | -0.0965 | Yes |
| 157 | MDK | na |  |  | 19037 | -2.689 | -0.0790 | Yes |
| 158 | WISP2 | na |  |  | 19077 | -2.817 | -0.0616 | Yes |
| 159 | COX6C | na |  |  | 19125 | -3.037 | -0.0432 | Yes |
| 160 | CA12 | na |  |  | 19153 | -3.311 | -0.0218 | Yes |
| 161 | DYNLT3 | na |  |  | 19169 | -3.505 | 0.0015 | Yes |
Table: GSEA details [plain text format]

  

Fig 2: HALLMARK\_ESTROGEN\_RESPONSE\_LATE      
 Blue-Pink O' Gram in the Space of the Analyzed GeneSet

  

Fig 3: HALLMARK\_ESTROGEN\_RESPONSE\_LATE: Random ES distribution      
 Gene set null distribution of ES for **HALLMARK\_ESTROGEN\_RESPONSE\_LATE**
